# New pairings and deorphanization among the atypical chemokine receptor family — physiological and clinical relevance

**DOI:** 10.3389/fimmu.2023.1133394

**Published:** 2023-04-20

**Authors:** Martyna Szpakowska, Giulia D’Uonnolo, Rafael Luís, Ana Alonso Bartolomé, Marcus Thelen, Daniel F. Legler, Andy Chevigné

**Affiliations:** ^1^Immuno-Pharmacology and Interactomics,Department of Infection and Immunity, Luxembourg Institute of Health, Esch-sur-Alzette, Luxembourg; ^2^Faculty of Science, Technology and Medicine, University of Luxembourg, Esch-sur-Alzette, Luxembourg; ^3^Tumor Immunotherapy and Microenvironment, Department of Cancer Research, Luxembourg Institute of Health, Luxembourg City, Luxembourg; ^4^Faculty of Biomedical Sciences, Institute for Research in Biomedicine, Università della Svizzera italiana, Bellinzona, Switzerland; ^5^Biotechnology Institute Thurgau (BITg) at the University of Konstanz, Kreuzlingen, Switzerland

**Keywords:** ACKR1, ACKR2, ACKR3, ACKR4, ACKR5, D6, CXCR7, GPR182

## Abstract

Atypical chemokine receptors (ACKRs) form a small subfamily of receptors (ACKR1–4) unable to trigger G protein-dependent signaling in response to their ligands. They do, however, play a crucial regulatory role in chemokine biology by capturing, scavenging or transporting chemokines, thereby regulating their availability and signaling through classical chemokine receptors. ACKRs add thus another layer of complexity to the intricate chemokine–receptor interaction network. Recently, targeted approaches and screening programs aiming at reassessing chemokine activity towards ACKRs identified several new pairings such as the dimeric CXCL12 with ACKR1, CXCL2, CXCL10 and CCL26 with ACKR2, the viral broad-spectrum chemokine vCCL2/vMIP-II, a range of opioid peptides and PAMP-12 with ACKR3 as well as CCL20 and CCL22 with ACKR4. Moreover, GPR182 (ACKR5) has been lately proposed as a new promiscuous atypical chemokine receptor with scavenging activity notably towards CXCL9, CXCL10, CXCL12 and CXCL13. Altogether, these findings reveal new degrees of complexity of the chemokine network and expand the panel of ACKR ligands and regulatory functions. In this minireview, we present and discuss these new pairings, their physiological and clinical relevance as well as the opportunities they open for targeting ACKRs in innovative therapeutic strategies.

## 1 Introduction

Chemokines (or chemotactic cytokines) are small soluble proteins (8–14 kDa) that guide cell migration and orchestrate several vital processes, including leukocyte recruitment during immunosurveillance. They are also involved in numerous inflammatory diseases and the development and spread of many cancers (1). They act through classical chemokine receptors (CKRs) that belong to the seven-transmembrane domain G protein-coupled receptor (GPCR) superfamily. Functionally, chemokines can be categorized as homeostatic or inflammatory according to their properties. Structurally, based on specific cysteine motifs in their N termini they are classified as CC, CXC, XC and CX_3_C chemokines and their receptors are consequently named CCR, CXCR, XCR and CX_3_CR (2).

**Figure 1:**
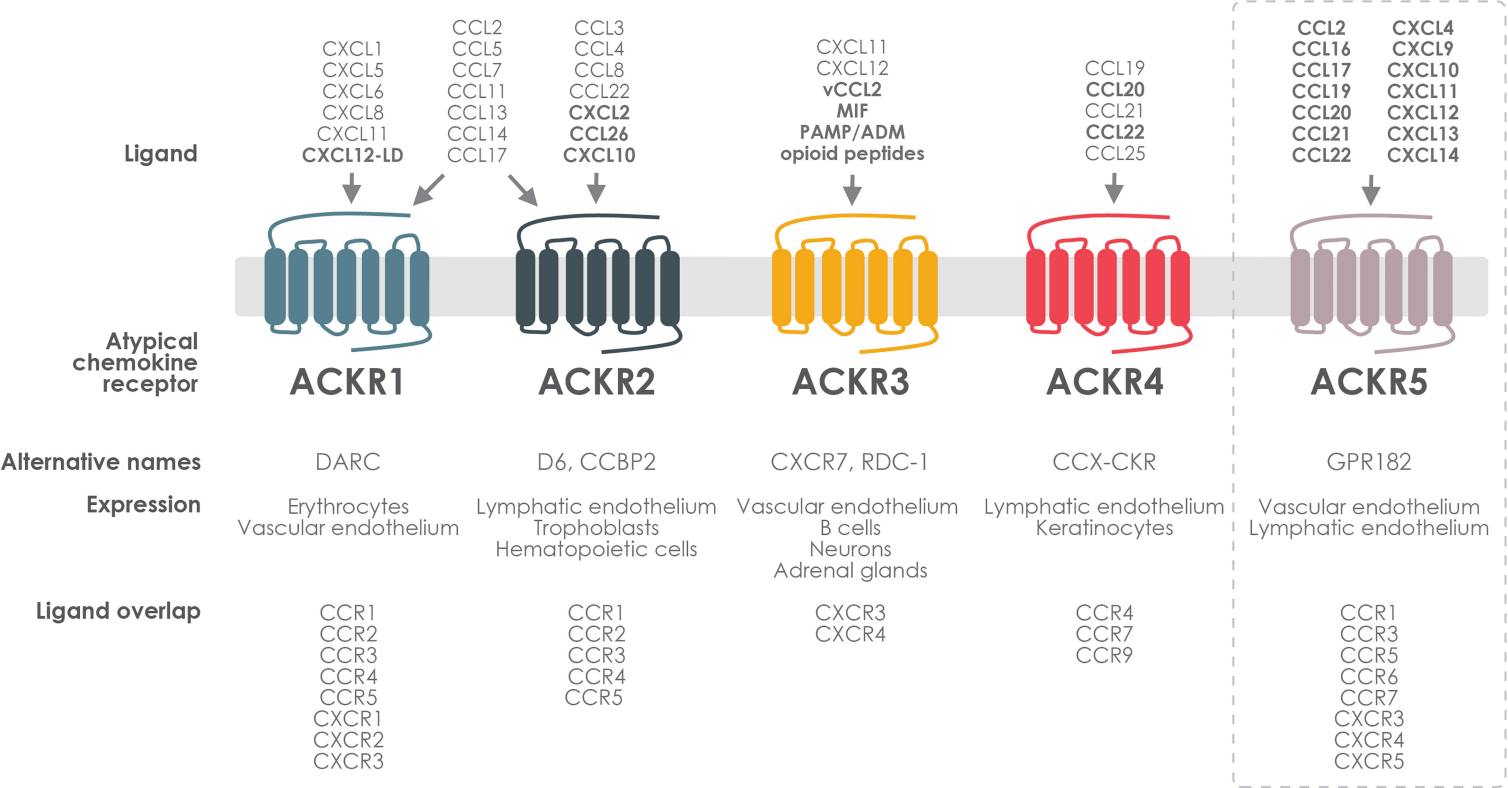
ACKR expression, ligand selectivity and crosstalk with classical chemokine receptors. Atypical chemokine receptors are expressed on different types of endothelial or immune cells. ACKR1 and ACKR2 bind a broad spectrum of inflammatory chemokines that they share with CXCR1–3 and CCR1–5. ACKR3 binds the homeostatic chemokine CXCL12, which it shares with CXCR4, and the inflammatory CXCL11, shared with CXCR3. ACKR3 also binds MIF and small non-chemokine peptides such as the proadrenomedullin-derived peptides, ADM and PAMP, as well as several opioid peptides. ACKR4 interacts with a limited number of mainly homeostatic chemokines that it shares with CCR4, CCR7 and CCR9. ACKR5 binds a wide range of both CC and CXC chemokines shared with CCR1, CCR3, CCR5–7 and CXCR3–5 and is still awaiting official IUPHAR recognition as an atypical chemokine receptor (dashed rectangle). Newly identified pairings are indicated in bold. CXCL12–LD: CXCL12 locked dimer.

Over the past years, an important subfamily of chemokine receptors has emerged as key regulators of chemokine functions. Formerly named chemokine-binding proteins, decoys, scavengers or interceptors, the standard nomenclature for this membrane protein family is now atypical chemokine receptors (ACKRs) (3, 4) (**Figure 1**). ACKRs are generally expressed on lymphatic and vascular endothelium, the epithelium of barrier organs and to a lesser extent on circulating leukocytes, in contrast to the classical chemokine receptors that are mainly found on hematopoietic and immune cells (5, 6). Although ACKRs form a rather diverse group and do not cluster phylogenetically, they do share several characteristics. Among their main common features is the inability to trigger the canonical G protein-mediated signaling or to directly induce cell migration in response to chemokines. Despite this atypicality, ACKRs fulfill essential regulatory functions in the chemokine–receptor network. Their well-established role is the tight regulation of chemokine concentration, for instance in inflammatory processes, and the formation of chemokine gradients for the signaling chemokine receptors, which is accomplished by the capture, transport or internalization of chemokines into degradative compartments or their presentation on cells (4, 7, 8). Other distinctive properties of ACKRs are their unconventional cellular localization, trafficking and expression profile. Indeed, most ACKRs are predominantly found in endosomal vesicles and several can cycle constitutively between the plasma membrane and the intracellular compartments, efficiently scavenging the bound chemokines (3, 7, 9–11). Although these functions were previously considered to mainly rely on β-arrestins, recent reports showed that they are not indispensable (12–17). Dimerization with canonical receptors and consequent alteration of expression and signaling properties is another characteristic of ACKRs that allows modulation of the chemokine network (8, 18, 19).

To date, out of the 23 chemokine receptors recognized by the International Union of Basic and Clinical Pharmacology (IUPHAR), four are members of the ACKR family (ACKR1–4) (20). This group of atypical receptors will presumably increase in the near future, both in terms of number and relevance. Indeed, for each of the ACKRs, recent pairings with chemokines or, as in the case of ACKR3 non-chemokine ligands, have been reported, and it is expected that new members, such as the recently deorphanized promiscuous chemokine scavenger GPR182 (ACKR5), will further enlarge this family.

In this minireview, we present and discuss these new pairings, their physiological and clinical relevance but also the growing number of properties that unify this somewhat heterogeneous receptor subfamily.

## 2 Pairing of dimeric CXCL12 with ACKR1

ACKR1 (formerly DARC for Duffy Antigen Receptor for Chemokines) is the oldest known chemokine receptor. It is barely recognizable as one from its primary amino acid sequence and its phylogenetic association (21, 22) and was initially described as blood group antigen and as a receptor for the Duffy Binding Proteins (DBP) from *Plasmodium knowlesi* and *Plasmodium vivax* malaria parasites (23–25). ACKR1 is prominently expressed on erythrocytes and venular endothelial cells, but not on capillaries or arteries (26–28). ACKR1 owes its distinctive regulatory function to its ability to internalize chemokines in polarized cells, mediating their transcytosis and increasing their bioavailability by presenting bound chemokines to other chemokine receptors in a spatiotemporally well-defined manner (29). Although ACKR1 is unable to promote the degradation of its ligands, it can compete with classical receptors for chemokine binding or reduce their availability in defined regions via internalization. By this mechanism, ACKR1 was proposed to play a role in impairing chemokine-induced angiogenesis (30, 31). On erythrocytes, ACKR1 binds circulating inflammatory chemokines with high affinity and can act as a “sink” or as a “buffer”. Indeed, a number of studies showed that ACKR1 modulates inflammatory responses by depleting its ligands (32, 33).

ACKR1 is the most promiscuous chemokine receptor with over ten chemokine ligands from the CC and CXC chemokine families (34–36). Studies carried out in the 1990s identified several chemokine ligands for ACKR1, which included CXCL1, CXCL4, CXCL7, CXCL8, CCL5, and CCL2 (34, 37). Since then, many more have been discovered with a broad range of affinities. Among the additional chemokines, CXCL2, CXCL3, CXCL5, CXCL6, CXCL7, CXCL11, CCL7, CCL11, CCL13, CCL14 and CCL17 exhibit strong binding to ACKR1 (36). Most of ACKR1 ligands are classified as inflammatory chemokines, with the receptor exhibiting no preference for either CC or CXC chemokines (36). In contrast, the majority of homeostatic and angiostatic ELR-chemokines show weak or no binding (36, 38, 39).

Recently, using biophysical analysis and immunofluorescence microscopy, ACKR1 was shown to bind with the dimeric form of CXCL12 (40). CXCL12 plays an important part in tissue development, vascular integrity, hematopoiesis, and immunity. Its effects through the interaction with the classical receptor CXCR4 and the atypical receptor ACKR3 have been studied extensively (41–43). It has now been suggested that ACKR1 promotes CXCL12 dimerization, which could potentially interfere with its monomeric signaling (44). The interaction between the CXCL12 dimer and ACKR1 suggests a potential new function for ACKR1 to modify the chemokine’s monomer–dimer equilibrium, further deepening the complexity of the functional regulation of CXCL12 (40).

## 3 Pairing of CXC and CC chemokines with the promiscuous CC chemokine scavenger ACKR2

ACKR2 (formerly D6 or CCBP2), identified in 1997, was until recently reported to exclusively bind inflammatory CC chemokines (45). The main ACKR2 ligands include CCL2–8, CCL11–14, CCL17 and CCL22, which are shared with the classical inflammatory receptors CCR1–5 (46–49). By scavenging these chemokines, ACKR2 is proposed to drive the resolution phase of inflammation and prevent exacerbated immune responses (50–55).

The pairing of ACKR2 with CC chemokines dates from when many chemokines, especially from the CXC class, were not yet known or readily available (45, 46, 49). A recent effort to systematically evaluate the activity of a full array of human and viral chemokines on ACKR2, by examining their ability to induce β-arrestin recruitment, revealed at least one more CC, CCL26, and two CXC chemokines, namely CXCL2 and CXCL10 as ligands of ACKR2 (56) with different potencies and efficacies (**Supplementary Figure 1**).

CCL26 was identified as a low-potency partial agonist of ACKR2, able to compete with other partial agonists for the binding and uptake by the receptor. CCL26 was previously demonstrated to bind and activate CCR3, although it has also been proposed as a ligand of CX_3_CR1 (57, 58). Though the functional relevance of the interaction between ACKR2 and CCL26 remains largely unknown, this chemokine–receptor pair may play a major role in a range of immune-mediated diseases. For instance, in persistent asthma, CCL26 was shown as the most effective inducer of eosinophil migration (59), while ACKR2, which is constitutively expressed in the lung, was shown to reduce airway reactivity by scavenging chemokines (60). Furthermore, considering ACKR2 was described to prevent spread of psoriasiform inflammation (61) and high serum levels of CCL26 were correlated with atopic dermatitis severity (62), it is possible that this new pairing will shed light on mechanisms of autoimmune inflammation. CXCL10, previously known to bind exclusively to CXCR3, is the strongest CXC chemokine identified activating ACKR2. CXCL10 was shown to act as a partial agonist of ACKR2 with potency in the low nanomolar range, inducing approximately half of the maximal response measured with its known full agonist CCL5. This partial agonist behavior was reminiscent of the activity towards its long-established signaling receptor CXCR3 relative to the full agonist CXCL11 (63, 64). Moreover, the potency of CXCL10 towards ACKR2 was approximately three times stronger than towards CXCR3. The rapid mobilization of ACKR2 to the plasma membrane induced by CXCL10 was similar to that observed in the presence of CC chemokines (65, 66), while imaging flow cytometry revealed specific and efficient uptake of labelled CXCL10 by ACKR2-expressing cells. Importantly, the ACKR2-driven intracellular accumulation of CXCL10 was also associated with a reduction of its availability in the extracellular space, pointing towards a regulatory role of ACKR2 for this CXC chemokine. Of note, CXCL10 is a pivotal inflammatory CXC chemokine in many physiological and pathological processes, including angiogenesis, chronic inflammation, immune dysfunction, tumor development and dissemination (67, 68), in which ACKR2 was also shown to be involved (6).

Noteworthy, CXCL2 also showed activity towards ACKR2, although it was weak in comparison to CXCL10 or to the activity it displays towards its classical receptor, CXCR2 (69–72). CXCL2 has no scavenger reported and is an important inflammatory chemokine and a powerful neutrophil chemoattractant. Interestingly, it has recently been reported that ACKR2-deficient mice show increased neutrophil infiltration in different tissues (73) and a higher anti-metastatic activity of neutrophils than normal mice (74). It remains to be investigated whether the enhancement of these neutrophil-related processes results from the suppression of CXCL2 regulation by ACKR2.

## 4 Pairing of a CC chemokine and non-chemokine endogenous peptides with ACKR3

ACKR3 (CXCR7 or RDC-1) is the second to last deorphanized chemokine receptor. It was initially shown to bind and be activated only by CXC chemokines, namely CXCL12 and CXCL11, which are also ligands for CXCR4 and CXCR3, respectively (41, 75). ACKR3 is expressed by endothelial cells, mesenchymal cells, B cells (76–78), in diverse regions of the central nervous system and in the adrenal glands (79–81). ACKR3-deficient mice die perinatally due to semilunar heart valve malformation and ventricular septal defects and show disrupted lymphangiogenesis and cardiomyocyte hyperplasia, despite no alterations in hematopoiesis (82, 83). Similarly to other scavenging receptors, ACKR3 is generally present intracellularly, and cycles continuously between the plasma membrane and the endosomal compartments (84–86). The scavenging function of ACKR3 was convincingly illustrated in studies using zebrafish embryos, where it shapes CXCL12 gradient during development (42, 87).

In 2018, a study demonstrated that the broad-spectrum antagonist CC chemokine vMIP-II/vCCL2 encoded by the sarcoma-associated herpesvirus (HHV-8) can bind and activate ACKR3 with potency somewhat lower than the endogenous CXC chemokines (88). ACKR3 scavenging of vCCL2 was proposed to impact the life cycle and immune escape of HHV-8 by controlling the availability of this important chemokine and its activity on both viral and host receptors. The identification of vCCL2 as a third chemokine ligand for ACKR3 and the first CC chemokine was also particularly valuable in the understanding of the activation mechanism and function of this atypical receptor (70).

ACKR3 was also shown to be the receptor for the pseudo-chemokine macrophage migration-inhibitory factor (MIF) (89). MIF is an inflammatory cytokine that functions as a chemoattractant and participates in innate and adaptive immune responses by promoting macrophage activation and B-cell survival (90–92). MIF is also a mediator in numerous inflammatory conditions and cancers (91, 93). MIF binding to ACKR3 was shown to promote receptor internalization and to contribute to cell signaling and B-cell chemotaxis (89). Moreover, MIF-induced ACKR3 signaling in platelets was described to modulate cell survival and thrombus formation (94).

Besides chemokines and pseudo-chemokines, ACKR3 was shown to bind several small peptide ligands. ACKR3 was proposed as a scavenger receptor for the two pro-angiogenic peptides adrenomedullin (ADM) and proadrenomedullin N-terminal 20 peptide (PAMP) (95) both encoded by the *Adm* gene, regulating their activity for the cognate receptors CLR/RAMPs and MgRX2, respectively (96, 97). These findings were in line with the observation that *Ackr3* knockout recapitulates the *Adm* overexpression phenotype and that silencing *Adm* expression counterweighs lymphatic and cardiac aberrations observed in *Ackr3* knockout mice (96). Nevertheless, the respective contribution of the two *Adm*-encoded peptides in the phenotype observed requires further investigation as ADM binds to ACKR3 at high micromolar concentrations, whereas processed forms of PAMP have potencies in the nanomolar range (95). ACKR3 was also shown to be a high-affinity scavenger for a broad spectrum of opioid peptides, especially enkephalins and dynorphins, binding and internalizing them. ACKR3 was thus proposed to reduce the availability of these peptides in important opioid centers in the central nervous system, where it is co-expressed with the classical opioid receptors. Modulation of the negative regulatory function of ACKR3 by molecules such as LIH383 or conolidine, an analgesic alkaloid used in traditional Chinese medicine, was shown to potentiate the activity of endogenous opioid peptides towards classical receptors, possibly opening alternative therapeutic avenues for opioid-related disorders (13, 98–101).

## 5 Pairing of the CC chemokines CCL20 and CCL22 with ACKR4

ACKR4 was deorphanized in 2000 (102). It was proposed to bind CCL19, CCL21, CCL25 and CXCL13, which are the ligands for CCR7, CCR9 and CXCR5, respectively (12, 102, 103). By scavenging these chemokines, ACKR4 was shown to regulate the trafficking and positioning of T cells and dendritic cells (104, 105). ACKR4 is best known for its role in shaping the gradient of CCL19 and CCL21 for CCR7-expressing dendritic cells in the subcapsular sinuses of the lymph nodes in the initial phase of the adaptive immune response (106, 107).

In a recent study, CCL20, previously known to bind exclusively CCR6, was identified as a novel ligand for ACKR4 (108). The authors predicted this chemokine–receptor pairing based on CCL20 sequence and expression similarities with CCL19 and CCL21. They demonstrated that CCL20 triggers β-arrestin recruitment to ACKR4, and is efficiently scavenged by ACKR4-expressing cells, both *in vitro* and *in vivo*. They proposed that by scavenging CCL20, ACKR4 regulates its availability for the classical receptor CCR6 and thereby plays a role in the positioning of CCR6-positive leukocytes within secondary lymphoid tissues for effective humoral and memory immune responses (108).

A parallel systematic pairing analysis using β-arrestin recruitment as readout confirmed CCL20 as a new full agonist ligand for ACKR4 with nanomolar potency (109). This study also found that CCL22 acts as a potent partial agonist of ACKR4. CCL22, which is a key player in both homeostasis and resolution of inflammatory responses was until then known for its ability to interact with CCR4 and ACKR2. Interestingly, in line with a previous report (110) this study also disproved the agonist activity of CXCL13 towards ACKR4 (109).

## 6 Deorphanization of GPR182/ACKR5 as a promiscuous scavenger receptor for both CC and CXC chemokines

Until very recently the G protein-coupled receptor 182 (GPR182, formerly known as ADMR) was classified as a class A orphan GPCR. Phylogenetically, it clusters within the chemokine receptor family owing to its 40% sequence similarity to ACKR3 (111). GPR182 was previously suggested as a receptor for adrenomedullin (112), which was later not confirmed (113). It was initially described to be present in several organs (80, 111), further studies identified its prevalent expression in endothelial cells in mouse and zebrafish (114), where it was proposed as a regulator of hematopoiesis.

In 2021, GPR182 was deorphanized and proposed as a new atypical chemokine receptor for CXCL10, CXCL12 and CXCL13 (115). The study confirmed the GPR182 expression in the endothelial compartment by using a transgenic mouse model expressing mCherry fluorescent protein under the control of mouse *Gpr182* promoter. GPR182 was detected in vascular endothelium of lungs, bone marrow, lymph nodes, Peyer’s Patches, liver and spleen but not in the vascular endothelium of conductive arterial vessel. It was also detected in lymphatic vessels from skin, intestine and lymph nodes. As its closest paralogue ACKR3, GPR182 was shown to bind CXCL12 with nanomolar affinity. CXCL10 was also a strong ligand for GPR182 and several other binders could be identified from a large set of human chemokines screened in binding competition studies with fluorescently labelled CXCL10, including CXCL13, CCL19 and CCL16.

More recently, a study highlighted GPR182 expression in lymphatic endothelial cells in human melanoma (116). In accordance with the first report, GPR182 was suggested as a novel atypical chemokine receptor for an extended spectrum of chemokines of different families and was tentatively named ACKR5. The authors primarily identified the CXCR3 ligand CXCL9 as able to bind GPR182. Competition binding studies with a set of 35 chemokines revealed the ability of GPR182 to interact also with the other CXCR3 ligands, CXCL10 and CXCL11 as well as promiscuous binding for chemokines belonging to the four different classes (CCL, CXCL, CX_3_CL and XCL). The authors suggested that GPR182 might be able to recognize GAG-binding motif, which is critical region for chemokines to adhere to the endothelium. Different GAG-biding peptides were able to disrupt CXCL9–GPR182 interaction, which led the authors to consider the GAG-binding motif as determinant for chemokine interaction.

Interestingly, both studies demonstrated the absence of Gprotein signaling in response to chemokine binding to GPR182 (115, 116), which is a common feature in the atypical chemokine receptor family (3, 4). Of note, a strong constitutive interaction with β-arrestin-2 was observed but no ligand-induced β-arrestin recruitment could be detected (115, 116). However, β-arrestins were suggested to be responsible for the rapid and spontaneous receptor internalization (115). An important scavenging ability was highlighted by rapid uptake of labelled chemokine in GPR182-expressing cells and the increased plasma levels of CXCL10, CXCL12 and CXCL13 in both full- and endothelial compartment GPR182 knockout mice (115). These mice also showed alteration in hematopoiesis, which is consistent with GPR182 scavenging of CXCL12 (115), a chemokine notably involved in this process (115, 117). Absence of GPR182 also determined increased intratumoral concentration of different chemokines (CCL2, CCL22, CXCL1, CXCL9 and CXCL10) (116), which was suggested to contribute to an increased recruitment of tumor infiltrating lymphocytes and, therefore, hypothesized as potential target for improved immunotherapy (116).

Further studies are needed to validate GPR182 ligand specificity, as this aspect is not entirely consistent between the two studies. Both studies do however propose GPR182 as a broad-spectrum atypical chemokine receptor. This is particularly interesting as it would represent the only scavenger receptors identified so far for chemokines like CXCL9, CXCL13, CCL16 and CCL28. In the absence of detectable ligand-induced GPR182 signaling, it is challenging to determine precisely the receptor selectivity as well as its molecular characterization. It renders the official inclusion of GPR182 in the atypical chemokine receptor family by the IUPHAR particularly complex.

## 7 Discussion

Significant progress has been made over the last decade towards a better comprehension of the functional and molecular aspects underlying the activity of ACKRs in health and disease. They have been gaining continuous consideration and are presently regarded as one of the most important receptor family standing at the forefront of the chemokine research and holding great therapeutic potential (6, 118–120).

The unifying characteristic of ACKRs and unique integration criteria is so far their inability to trigger G protein signaling in response to chemokine binding. However, ACKRs often share other properties, such as the predominant intracellular localization or the ability to constitutively cycle between the plasma membrane and the intracellular compartments. Furthermore, early and more recent pairings suggest that ACKRs are commonly responsive to chemokines from different families. Indeed, the ability to bind and respond to both CC and CXC chemokines was historically described for ACKR1 (121) and — although it was subsequently challenged (109, 110) — for ACKR4 (102). This cross-family selectivity has now been extended to ACKR2 (56), ACKR3 (88) and ACKR5 (115, 116) and therefore appears to represent an additional functional characteristic of ACKRs (3) that is not observed among the classical chemokine receptors.

Despite the many similarities, each ACKR presents its own distinct particularities in terms of expression pattern, ligand selectivity, function and mode of action. For instance, while most ACKRs interact with β-arrestins, ACKR1 seems to be an exception. ACKR3 also stands out in its atypicality as it is highly prone to activation (70) and can act as a receptor also for non-chemokine small peptide ligands (13, 95, 98). Whether these two properties are linked and exclusive to ACKR3 or shared with other ACKRs remains to be investigated. Finally, GPR182 (ACKR5) seems to be a highly promiscuous receptor continuously scavenging chemokines with high basal β-arrestin association (115, 116).

While it may seem surprising that several chemokine–ACKR pairings have been identified only recently, it was made possible thanks to different technological and scientific advances. For the long-established ACKRs, the better understanding of their function, mode of action and the commercial availability of chemokines as recombinant proteins have facilitated the recent pairings. Most importantly, the development of various sensitive assays allowing to accurately detect the activity of chemokines on the receptors independently of G protein signaling, e.g. via the induction of β-arrestin recruitment or the modification of the receptor trafficking or localization, have been instrumental to identifying new ligand–receptor interactions (122). In case of GPR182, which shows high level of basal cycling activity and β-arrestin interactions, a combination of experimental approaches allowed for its deorphanization. Receptor sequence comparison, precise determination of the expression profile and the use of binding competition studies confirmed by increased chemokine plasma concentration in knockout mice, were required to circumvent the problems related to the absence of direct chemokines-induced effects on the receptor (115, 116). For this receptor, additional independent investigations are now needed to precisely define the panel of chemokines it can scavenge and obtain an official inclusion by the IUPHAR in the ACKR family as ACKR5.

**Figure 2:**
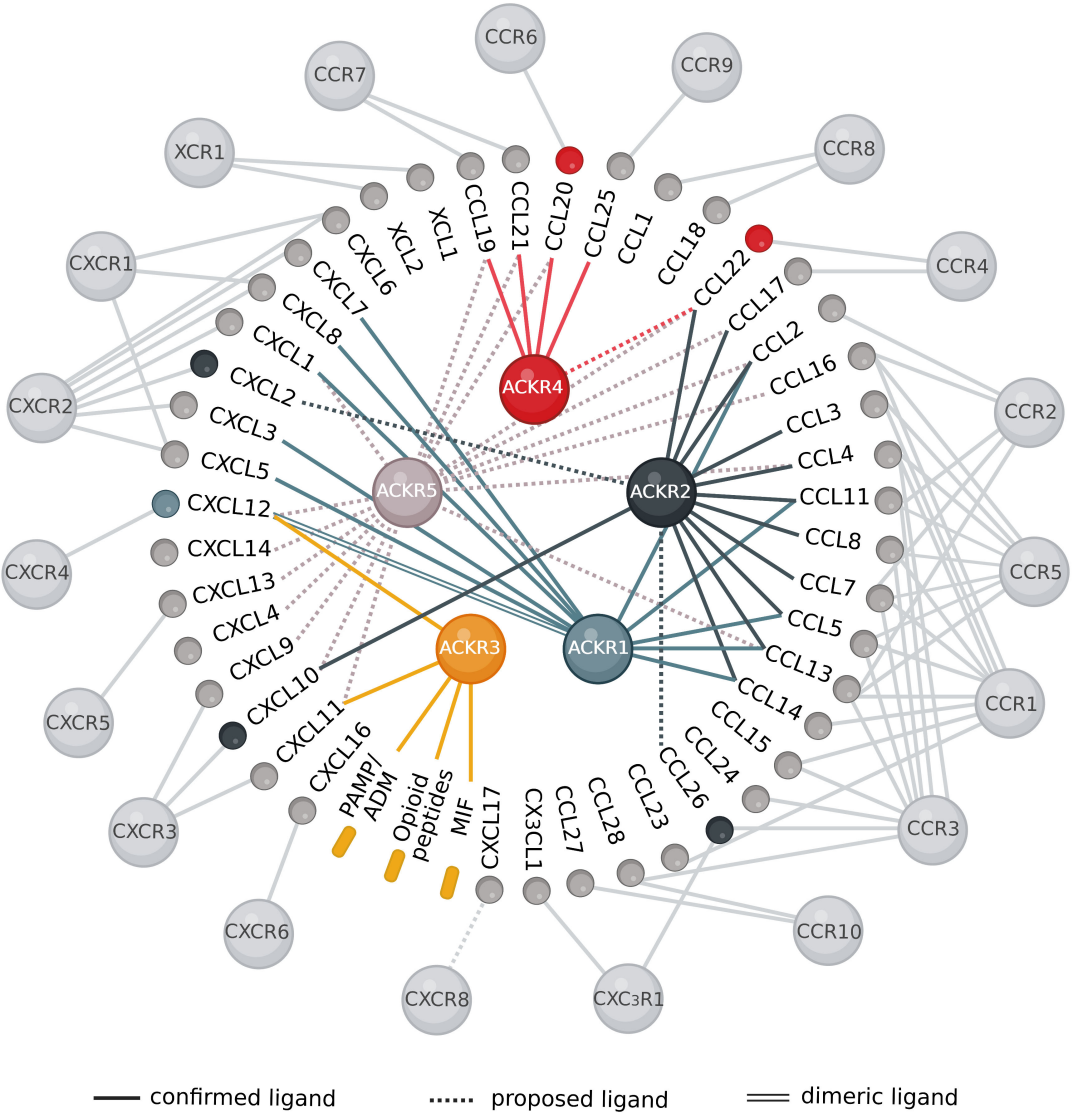
Overview of the chemokine interaction network with classical and atypical receptors. The interactions between different chemokines and their signaling and regulatory receptors are highly promiscuous. Most chemokines can bind several receptors and the majority of the receptors have multiple ligands. Receptors and chemokines are represented as spheres, while non-chemokine ligands are represented as rounded rectangles. There are 45 chemokines, 19 classical chemokine receptors (light grey) and 5 atypical chemokine receptors: ACKR1 (light blue), ACKR2 (dark blue), ACKR3 (yellow), ACKR4 (red) and the newly proposed ACKR5/GPR182 (light grey). Colored chemokines and non-chemokine ligands represent recently identified pairings, dashed lines indicate proposed ligands and double lines designate the binding of the dimeric ligand to the receptor. Created with BioRender.com.

The chemokine–receptor network is well recognized for its highly intricate interactions where a chemokine may interact with several receptors, while a chemokine receptor has usually multiple ligands (**Figure 2**). On the other hand, some chemokines may be exclusive of a single classical receptor. However, the recent pairings described above identified at least one ACKR for a number of these chemokines, such as CCL20 (CCR6), CCL25 (CCR9), CXCL2 (CXCR2), CXCL9 and CXCL10 (CXCR3), CXCL13 (CXCR5) expanding the panel of ACKR ligands and functions. To date, out of the 45 human chemokines, several of them binding to XCR1 (XCL1 and XCL2), CCR8 (CCL1 and CCL18), CCR10 (CCL27 and CCL28), CCR3 (CCL15 and CCL24), CCR1 (CCL23), CXCR1 (CXCL6), CXCR6 (CXCL16), CX_3_CR1 (CX_3_CR1) and the orphan chemokine CXCL17 have not been paired with an ACKR (**Figure 2**).

The recent new pairings suggest that a systematic reassessment of chemokine–receptor interactions for ACKRs but also long-established classical chemokine receptors may still be necessary. Indeed, owing to the functional selectivity and biased signaling reported for some chemokines and receptors, the attempts to uncover new pairings should not be limited to monitoring G protein signaling or β-arrestin recruitment. Other approaches such as measuring fluorescent ligand uptake, receptor trafficking or chemokine degradation in both agonist and antagonist modes should also be considered, as important crosstalks may remain unexplored.

The novel pairings among ACKRs add an unforeseen level of complexity to their functions and regulatory roles for chemokines and non-chemokine ligands, while they also open interesting therapeutic opportunities, notably for cancer and chronic pain. For instance, the identification of ACKR2 and GPR182 as scavenger receptors for CXCL10 and/or CXCL9, in addition to their well-established inflammatory CC chemokine ligands such as CCL2, CCL4 and CCL5, may be exploited in approaches seeking to turn cold tumors to hot tumors to improve the effectiveness of immunotherapies. Indeed, these newly identified chemokines for ACKR2 and GPR182 are key players in driving NK cells and CD8+ T cells into the tumor bed (123–126). Therefore targeting their receptors may consequently increase the chemokine levels in the tumor microenvironment and subsequently sensitize them to immunotherapy (56, 118). On the other hand, targeting ACKR3 and blocking its proposed opioid peptide scavenging function was proposed as a new avenue to develop safer drugs with less side effects, which is critically needed to treat chronic pain (100, 101).

However, considering the importance and multiplicity of their functions, the constantly growing number of ligands identified, the complexity of their biology and the interconnectivity with multiple systems, the targeting of ACKRs remains a great challenge. So far, only small molecules, peptides, modified chemokines and antibody fragments targeting ACKR3 have been reported, partly owing to the long-established importance of the CXCR4–CXCL12 axis in cancer, autoimmune and cardiovascular diseases (13, 70, 119, 120, 127–131). Nevertheless, the increasing number of studies showing implication of other ACKRs, including ACKR5, in cancer development, progression but also protection together with the increasing availability of screening assays specific for each ACKR will likely favor in the new future the development of modulators for other members of the family (100, 122).

In the coming years, the ACKR family may be further enlarged (132). Indeed, CXCR3B, the extended isoform of CXCR3, was recently proposed to display attributes of ACKRs (133), while CCRL2 and PITPNM3 await validation with regard to chemokine binding and direct regulatory functions (134–136). Additional studies will reveal whether the latter two share common functional properties with the established and newly deorphanized atypical chemokine receptors.

In summary, investigations on ACKR are still in a highly dynamic phase and the recent identification of new pairings for established members of the family and of GPR182 as new member will certainly reinforce the interest of the community for this fascinating class of receptors. A better understanding of their functional complexity and heterogeneity is still needed in light of the extended panel of ligands they regulate and the therapeutic potential they seem to hold.

## Author contributions 

MS and AC designed the manuscript. MS, GD’U, RL, AAB, and AC wrote the manuscript. MS, GD’U, RL, MT, DL, and AC revised the manuscript. All authors contributed to the article and approved the submitted version.
