# Supplementary material for: New pairings and deorphanization among the atypical chemokine receptor family — physiological and clinical relevance
Source: Front Immunol. 2023 Apr 20;14:1133394. doi: 10.3389/fimmu.2023.1133394 (PMC10157204; doi:10.3389/fimmu.2023.1133394)
Supplement: Supplementary file 1 [file DataSheet_1.docx]

**
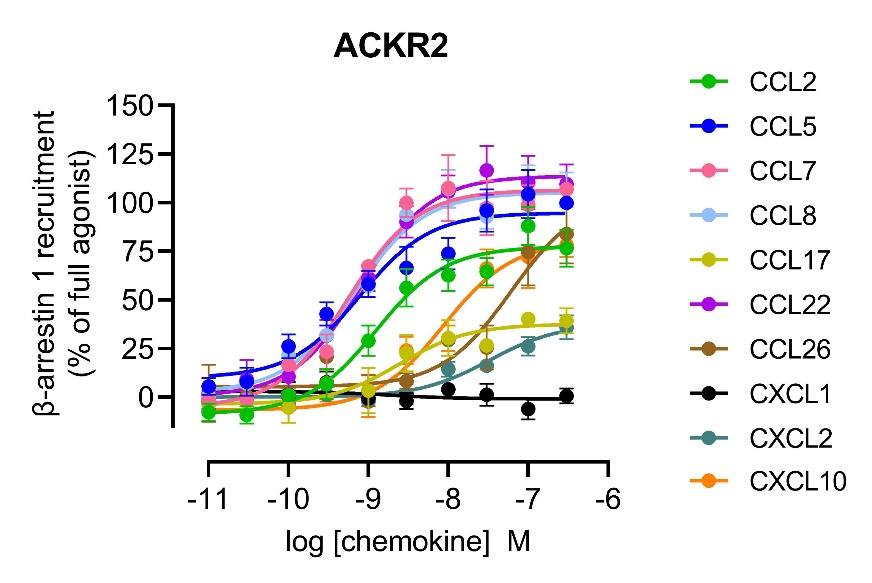
**

**Supplementary Figure 1.** **ACKR2 activation by different ligands.** β-arrestin-1 recruitment to ACKR2 induced by known and the newly proposed ligands such as CCL26, CXCL2 and CXCL10 monitored by Nanoluciferase complementation assay (NanoBiT). CXCL1 was used as negative control. The assays were conducted in HEK293T cells. Data points represent mean ± SEM of three independent experiments.
